# Psychometric Validation of the Bangla Version of the Patient-Doctor Relationship Questionnaire

**DOI:** 10.1155/2016/9385364

**Published:** 2016-01-21

**Authors:** S. M. Yasir Arafat

**Affiliations:** Department of Public Health, ASA University Bangladesh, ASA Tower, 23/3 Bir Uttam ANM Nuruzzaman Sarak, Shyamoli, Dhaka 1207, Bangladesh

## Abstract

*Background*. The patient-doctor relationship is an important issue in health care as it is linked to patient satisfaction, treatment adherence, and treatment outcome. The PDRQ-9 is brief instrument which has an excellent overall internal consistency to measure it.* Objective*. It was aimed at developing a culturally adapted and validated Bangla version of Patient-Doctor Relationship Questionnaire (PDRQ-9).* Method*. Data were collected during the period of May 2015 to July 2015 from 50 patients by interviewing with the final Bangla version of PDRQ-9 obtained by ideal translation-back translation procedure with nonprobability purposive consecutive sampling and analyzed by Statistical Package of Social Science (SPSS) 16.0 and Microsoft Excel 2007 version software.* Result*. The internal consistency of Bangla PDRQ-9 was measured by Cronbach's *α* which was 0.97. Only one factor was extracted from varimax rotation factor analysis with high commonalities between the items.* Conclusion*. Bangla version of PDRQ-9 is valid, accepted, and widely applicable in clinical practice, research, public health, and primary health care in Bangladesh.

## 1. Introduction

The patient-doctor relationship (PDR) is an important concept in health care [1]. Throughout the history of medicine, patients and doctors have scrutinized and debated their relationship, which is undoubtedly one of the most difficult among interpersonal relations, for a variety of reasons [2]. It has been linked to patient satisfaction, treatment adherence, and treatment outcome. In primary care, “knowing the patient is at least as important as knowing the disease,” and physicians with a warm and friendly style are more effective than physicians with a more formal style [3]. It was found that patients search out a Primary Care Practitioner (PCP) who matches their own representation of an ideal and validate their choice in a dynamic communicative process [4]. Patient satisfaction is a concept that reflects the patient's perception regarding the care quality and treatment received. Generally evaluated by self-report, several questionnaires focused on decision making, access, and use of the different health services or on the treatment satisfaction [5]. The measurement instruments of the patient-doctor relationship make it possible to quantify the patient's opinion regarding communication, satisfaction, and accessibility in the dealing with the doctor and the treatment followed [5]. Substantial efforts have been made to develop instruments and to assess the PDR from the patient's point of view. A systematic review found 19 instruments with a variety of dimensions and used diverse conceptual models for the PDR and recommended the use of the Patient-Doctor Relationship Questionnaire (PRDQ-9) as a brief (9 items) questionnaire with excellent overall internal consistency [1]. Bengali is one of the sixth most widely spoken languages in the world with nearly 300 million users. In 2050 estimated Bengali speaking population will be nearly 400 million [6]. At present there is no culturally adapted scale to measure the patient doctor relationship in Bangla. So, it was aimed at developing a culturally adapted and validated Bangla version of Patient-Doctor Relationship Questionnaire (PDRQ-9) for use in assessing the relationship between the physician and the patient as PDRQ-9 is brief short easily applicable tool.

## 2. Materials and Methods

### 2.1. Ethical Consideration

The researcher was duly concerned about the ethical issues relate to the study. Formal ethical clearance was taken from the ethical review committee of the ASA University Bangladesh for conducting the study and formal permission was taken from the questionnaire developing author, Christina M. Van der Feltz-Cornelis. Confidentiality of the person and the information was maintained and observed and unauthorized persons did not have any access to the data. Informed written consent was taken from the subject informing the nature and purpose of the study, the procedure of study, and the right to refuse, accept, and withdraw to participate in the study and the participants did not gain financial benefit from this study. The present study posed a very low risk to the participants, as procedures such as medical treatments, invasive diagnostics, or procedures causing psychological, spiritual, or social harm were not included.

### 2.2. Instrument Development

The adaptation to Bengali was performed according to the state-of-the-art procedure of forward-backward translation of English PDRQ-9 by 1 medical doctor and 1 psychologist; both of them are native speakers of the Bangla and are fluent in English. The 2 Bangla versions were compared, and an updated Bangla forward version was compiled. This version was translated back into English by a professional translator with experience in medical translation and by 1 medical doctor who had not been involved in the forward translation. The back-translated versions were then compiled and compared by the researcher. These versions were submitted to the University expert committee. Thus, an optimized Bangla version was generated and with that optimized Bangla version pretesting was done and supposed changes were made accordingly. Thus, the final Bangla version of PDRQ-9 was developed [7].

### 2.3. Design and Subjects

The current study was a part of Master of Public Health (M.P.H.) thesis under the department of Public Health, ASA University Bangladesh. The descriptive, cross-sectional study was conducted at Psychiatry Out-Patient Department (OPD) of Dhaka Medical College Hospital (DMCH) and data were collected during the period of May 2015 to July 2015 from 50 patients with the final Bangla version of PDRQ-9 with nonprobability purposive consecutive sampling. The interview was performed by the researcher himself through face-to-face interview technique. The participants who were above the age of 14 years, were willing to participate in the study, and can read Bangla were included in the study. Participants not willing to participate and who made first visit to the respective physicians were excluded. Sample size was estimated on basis of more than 5 : 1 ratio of participants to item as variable recommendations available considering rules of thumb regarding the minimum ratio of participants to variables 2 : 1 or extracted factors 20 : 1 [8]. The questionnaire encompasses nine statements and the respondents were requested to indicate the appropriateness of each statement as a description of how they feel about their doctor in different aspects. After managing data properly it was analyzed in SPSS 16 version and Microsoft Excel Software 2007 version.

## 3. Result

### 3.1. Demographic Characteristics of Respondents

In this PDRQ-9 Bangla validation study age, gender, educational status, occupation, religion, marital status, net family income, residence, family type, number of family members, and duration of follow-up were considered as demographic variables. Age of the respondents was found as mean ± SD (range): 35.6 ± 10.71 (14–66) years; 62% were male and 38% were female; 64% live in town, 16% in mini town, and 20% in village; 72% were married, 16% unmarried, 8% widow, and 4% divorced; 52% had nuclear family and 48% belonged to joint family environment.

Mean PDRQ-9 score of individual item ranged from 3.02 ± 1.11 to 3.30 ± 1.11. The highest score was 3.30 ± 1.11 for item-6 “Agreeness on the nature of symptoms.” The lowest score was 3.02 ± 1.11 for item-9 “Accessibility” (Table 1).

### 3.2. Reliability of PDRQ-9 Bangla

Cronbach's Alpha (*α*) value was 0.97. (When interpreting Cronbach's Alpha (*α*), it ranges from 0 to 1. A value of ≥0.70 reflects good reliability.)

Interitem correlation matrix showed a value of significant correlation (Table 2). (When interpreting correlation, it ranges from 0 to 1. A value of ≥0.50 reflects the items are significantly correlated.)

### 3.3. Validity of Bangla Version of PDRQ-9

*Face validity*,* content validity,* and* criterion validity* was systematically assessed and maintained during the development of the research instrument and at the time of interview by the interview response. Construct validity was assessed, done by Exploratory Factor Analysis, the principal component with varimax rotation and internal consistency. KMO and Bartlett's test of sphericity was applied to the fitness of data for factor analysis and the KMO and Bartlett's Test of sampling adequacy was 0.9 where a value of ≥0.5 of KMO is considered as a good sampling adequacy.

### 3.4. Construct Validity

Construct validity was assessed by factor analysis. It showed high commonalities between the items before and after extraction ranging from 0.79 to 0.85. The varimax rotation showed only one component was extracted (Table 3).

## 4. Discussion

Psychometrics has a very important role in public health, psychiatry, primary health care, and many other fields even in health promotional strategy for measuring the attitude. Relationship between patient and physician has been discussed from the origin of medicine. PDRQ-9 was developed by Van Der Feltz-Cornelis et al. in 2004, a Patient-Doctor Relationship Questionnaire (PDRQ-9) in primary care based on Alexander and Luborsky developed the Helping Alliance Questionnaire (HAQ), and Van der Linden validated Dutch version [4]. Spanish version was validated in 2009 by Adán et al., validation of the Turkish Patient-Doctor Relationship Questionnaire (PDRQ- Turkish) was done by Mergen et al. in 2012, and Zenger et al. validated the German version of PDRQ-9 in 2014 [1, 5, 9]. It was aimed at adapting the PDRQ-9 in Bangla.

Internal consistency of PDRQ-9 Bangla was found to be 0.97 (Cronbach's Alpha) which suggests significant reliability and aligns with PDRQ-9 German (0.95), PDRQ-9 Dutch (0.94), Spanish (0.92), and Turkish (0.91) validation study [1, 4, 5, 9]. The corrected item-total correlation was ≥0.87 (Table 2) which was highly significant and aligns with others as it was ≥0.94 in PDRQ-9 German validation study [1]. Factor analysis of PDRQ-9 Bangla was revealed as a single factor scale and the solution could not be rotated in the varimax rotation (Table 3) as PDRQ-9 German was unidimensional scale and PDRQ-9 Dutch, PDRQ Spanish were validated as single factor scale after removal of second factor with the help of the factor analysis without significant alteration of scale property, reliability, and validity ultimately [1, 4, 5].

## 5. Conclusion

The results from this evaluation indicate good psychometric properties of the PDRQ-9 Bangla. This Bangla validated scale will be helpful to assess the relationship between physician and patients in clinical practice, research, public health, and primary health care in Bangladesh. Though it is the first validated scale in Bangla for measuring the patient-doctor relationship and a very good scale to measure the relationship in a very short time in clinical practice, public heath, health research, and primary health care, with this minimum sample, generalization of the study result may be difficult. Data were collected from the patients visiting a hospital of a city, but those from hospitals or institutions of other regions and heterogeneous groups were not included. This instrument bears only the patients' direction of the relationship; doctors' direction was not to be evaluated. Larger studies involving more heterogeneous patients may help to provide a more complete picture of impact of patient-doctor relationship in Bangladesh.

## Figures and Tables

**Table 1:** Distribution of item characteristics of PDRQ-9 Bangla (*N* = 50).

Item number	PDRQ-9 item	Mean ± SD	Corrected item-total correlation
1	My physician helps me	3.06 ± 1.03	0.88
2	My physician has enough time for me	3.20 ± 1.17	0.89
3	I trust my physician	3.22 ± 1.09	0.87
4	My physician understands me	3.14 ± 1.22	0.88
5	My physician is dedicated to help me	3.10 ± 1.07	0.88
6	My physician and I agree on the nature of my medical symptoms	3.30 ± 1.11	0.86
7	I can talk to my physician	3.22 ± 1.18	0.92
8	I feel content with my physician's treatment	3.24 ± 1.22	0.87
9	I find my physician easily accessible	3.02 ± 1.11	0.90

**Table 2:** Interitem correlation of PDRQ-9 Bangla.

Interitem correlation matrix									
q1									
q2	.79								
q3	.85	.77							
q4	.76	.82	.70						
q5	.89	.80	.88	.73					
q6	.72	.81	.70	.92	.69				
q7	.85	.87	.87	.80	.83	.80			
q8	.79	.77	.80	.86	.79	.83	.81		
q9	.82	.86	.79	.83	.81	.81	.86	.76	

**Table 3:** Factor Analysis-Principal Component Analysis with distribution of varimax rotation of PDRQ-9 Bangla.

Rotated component matrix	
Items	Component
	1
q1	.91
q2	.91
q3	.90
q4	.90
q5	.90
q6	.89
q7	.94
q8	.90
q9	.92

## References

[1] Zenger M., Schaefert R., van der Feltz-Cornelis C., Brähler E., Häuser W. (2014). Validation of the patient-doctor-relationship questionnaire (PDRQ-9) in a representative cross-sectional German population survey. *PLoS ONE*.

[2] Koutsosimou M., Adamidis K., Liakos A., Mavreas V. (2013). The development of an instrument for the assessment of doctor-patient relationship (dopraq-16). *Journal of Psychology & Psychotherapy*.

[3] Eveleigh R. M., Muskens E., Van Ravesteijn H., Van Dijk I., Van Rijswijk E., Lucassen P. (2012). An overview of 19 instruments assessing the doctor-patient relationship: different models or concepts are used. *Journal of Clinical Epidemiology*.

[4] Van Der Feltz-Cornelis C. M., Van Oppen P., Van Marwijk H. W. J., De Beurs E., Van Dyck R. (2004). A patient-doctor relationship questionnaire (PDRQ-9) in primary care: development and psychometric evaluation. *General Hospital Psychiatry*.

[5] Adán J. C. M., Moreno-Jiménez B., Carvajal R. R., Herrer M. G., López P. R. (2009). Psychometric validation of the spanish version of the patient-doctor relationship questionnaire (PDRQ). *Actas Espanolas de Psiquiatria*.

[6] Chowdhury S. R. (2014). *Adaptation, Linguistic & Clinimetric Validation of the Bangla Version of the Brief Psychiatric Rating Scale (BPRS-18) for Bangladesh Index*.

[7] Beaton D. E., Bombardier C., Guillemin F., Ferraz M. B. (2000). Guidelines for the process of cross-cultural adaptation of self-report measures. *Spine*.

[8] Ridd M. J., Lewis G., Peters T. J., Salisbury C. (2011). Patient-doctor depth-of-relationship scale: development and validation. *Annals of Family Medicine*.

[9] Mergen H., van der Feltz-Cornelis C., Karoglu N., Mergen B. E., Ongel K. (2012). Validity of the Turkish patient-doctor relationship questionnaire (PDRQ-Turkish) in comparison with the Europe instrument in a family medicine center. *HealthMED Journal*.

